# Oxytocin receptor polymorphism influences characterization of harm avoidance by moderating susceptibility to affectionless control parenting

**DOI:** 10.1002/brb3.2393

**Published:** 2021-10-17

**Authors:** Keisuke Noto, Akihito Suzuki, Toshinori Shirata, Yoshihiko Matsumoto, Haruka Muraosa, Kaoru Goto, Koichi Otani

**Affiliations:** ^1^ Department of Psychiatry Yamagata University School of Medicine Yamagata Japan; ^2^ Department of Anatomy and Cell Biology Yamagata University School of Medicine Yamagata Japan

**Keywords:** oxytocin, parenting, personality, polymorphism, receptor

## Abstract

**Introduction:**

Oxytocin receptor (OXTR) gene polymorphism reportedly moderates effects of negative environments during childhood on mental function and behavior such as depressive symptoms and externalizing problems. This study examined OXTR gene polymorphism effects on personality traits in healthy participants, considering interaction effects of polymorphism with affectionless control (AC) parenting which is one of the dysfunctional and pathogenic parenting styles.

**Methods:**

For 496 Japanese volunteers, personality was evaluated using the Temperament and Character Inventory. The Parental Bonding Instrument, which has subscales of care and protection, was used to assess perceived parental rearing. AC parenting was defined as low care and high protection. A/G polymorphism of the OXTR gene (rs53576) was detected using TaqMan SNP Genotyping Assay.

**Results:**

Two‐way analysis of covariance revealed significant interaction effects between the genotype and the number of AC parents on scores of harm avoidance, with no significant main effect of genotype on any personality. Post‐hoc analysis revealed that the harm avoidance scores were increased in a stepwise manner with respect to the increase of the number of AC parents in the A allele carriers. No similar association was observed in the A allele noncarriers.

**Conclusion:**

The results of this study suggest that OXTR polymorphism influences characterization of harm avoidance by moderating susceptibility to AC parenting.

## Graphical abstract

This study examined oxytocin receptor (OXTR) gene polymorphism effects on personality traits in healthy participants, considering interaction effects of polymorphism with affectionless control (AC) parenting which is one of the dysfunctional and pathogenic parenting styles. Post‐hoc analysis revealed that the harm avoidance scores were increased in a stepwise manner with respect to the increase of the number of AC parents in the A allele carriers. The results of this study suggest that OXTR polymorphism influences characterization of harm avoidance by moderating susceptibility to AC parenting.

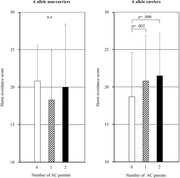

## 1 INTRODUCTION

Oxytocin, a neuropeptide synthesized in the supraoptic and paraventricular nuclei of the hypothalamus, uterus, testis, pancreas, and adrenal glands, is known to exert widely various peripheral and central effects such as reproductive systems, social and maternal behaviors, and stress‐associated responses (Gimpl & Fahrenholz, 2001). Physiological effects of oxytocin are mediated by binding to its specific receptors (oxytocin receptor [OXTR]), which are localized on human central and peripheral areas, that is, the cerebral cortex, limbic system, brainstem, uterus, ovary, and mammary glands (Gimpl & Fahrenholz, 2001).

The human OXTR gene, located at 3p25‐3p26.2, contains three introns and four exons (Gimpl & Fahrenholz, 2001; Kimura et al., 1992). Rs53576, an A/G single nucleotide polymorphism in the intron three of the OXTR gene, has been investigated extensively because of its association with human social behaviors (Cataldo et al., 2018). Two meta‐analyses using genetic association studies show that the A allele is associated with lower sociality (Li et al., 2015) and empathy (Gong et al., 2017). A neuroimaging study of healthy human volunteers revealed that the A allele of this polymorphism is associated with decreased hypothalamus volume, increased amygdala volume, and increased connection between the amygdala and hypothalamus during perceptual processing for facial emotion (Tost et al., 2010). Reportedly, the hippocampal volume is smaller in A allele carriers with high emotional trauma than in those with minimal emotional trauma, but no such relation was observed in A allele noncarriers (Malhi et al., 2020).

In relation to personality, effects of rs53576 polymorphism on personality traits have been examined (Cornelis et al., 2012; Saphire‐Bernstein et al., 2011; Tost et al., 2010; Wang et al., 2014) using the Tridimensional Personality Questionnaire (Cloninger et al., 1994), the Life Orientation Test (Scheier et al., 1994), and the Rosenberg Self‐Esteem Scale (Rosenberg, 1965). However, these studies produced inconsistent results related to harm avoidance (Tost et al., 2010; Wang et al., 2014) and optimism (Cornelis et al., 2012; Saphire‐Bernstein et al., 2011). Therefore, associations of this polymorphism with personality must be re‐examined. Furthermore, the results of recent studies particularly addressing the gene–environment interactions suggest that A allele carriers of the polymorphism are sensitive to childhood negative environments, leading to increased depressive symptoms (Kazantseva et al., 2020; Thompson et al., 2014) and behavioral problems (Choi et al., 2019). Nevertheless, no report has described a study examining effects of interaction between this polymorphism and environments on personality traits. Therefore, this study examined OXTR polymorphism effects on personality traits, as evaluated using the Temperament and Character Inventory (TCI; Cloninger et al., 1994), considering interaction effects of this polymorphism with affectionless control (AC) parenting which is a combination of poor‐care and over‐protection and is one of the dysfunctional and pathogenic parenting styles (Martin & Waite, 1994; Parker, 1979; Parker, 1983), as assessed using the Parental Bonding Instrument (PBI) (Parker et al., 1979).

## 2 METHODS

### 2.1 Participants

Participants were 496 healthy Japanese volunteers (305 male, 191 female, mean age ± SD (range) = 23.7 ± 3.1 (21–53) years) recruited from students and hospital staff members in Yamagata prefecture. Exclusion criteria were (1) participants with current or past history of psychiatric disorder according to the Diagnostic and Statistical Manual of Mental Disorders – IV (American Psychiatric Association, 1994) and (2) participants whose parents had divorced or died before the child was 16 years old. This study was performed in accordance with the Declaration of Helsinki. The Ethics Committee of the Yamagata University School of Medicine approved this study (approval no. 31/26.12.2002). Informed consent to this study was acquired in writing from all participants.

### 2.2 Assessment of personality and AC parenting

Personality traits of participants were evaluated using the Japanese version of the TCI, which has high validity and internal consistency (Cloninger et al., 1994; Kijima et al., 1996). The TCI has seven dimensions: harm avoidance, novelty seeking, reward dependence, persistence, self‐directedness, cooperativeness, and self‐transcendence.

Perceived parental rearing was assessed using the Japanese version of the PBI, which is verified to have high reliability and validity (Ogawa, 1981; Parker et al., 1979). The PBI has care and protection subscales from parents: paternal care, paternal protection, maternal care, and maternal protection. Each score was regarded as “low” if it was equal to or lower than the median, and “high” if it was higher than the median. AC parenting was inferred when participants had low care and high protection (Parker et al., 1979). Reportedly, AC parenting is a predisposing factor for neurotic depression in adulthood (Parker, 1979; Parker, 1983) and suicidality (Martin & Waite, 1994). Our earlier studies also demonstrate that AC parenting increases personality vulnerability to depression, such as interpersonal sensitivity (Otani et al., 2009) and dysfunctional attitude (Otani et al., 2014).

### 2.3 Genotyping

DNA was collected from peripheral blood using a QIAamp DNA Blood Kit (Qiagen, Tokyo, Japan). To detect the rs53576 of the OXTR gene, TaqMan SNP Genotyping Assay (C_3290335_20; Thermo Fisher Scientific) and TaqPath Master Mix (Thermo Fisher Scientific) were used. For this study, the OXTR genotypes were divided into two groups: A allele carriers (A/A + A/G) and A allele noncarriers (G/G), according to earlier meta‐analyses showing that the A allele is dominantly related to sociality (Li et al., 2015) and empathy (Gong et al., 2017).

### 2.4 Statistical analyses

Statistical analyses were conducted using software (SPSS 26; SPSS Japan Inc.). Two‐way analysis of covariance (ANCOVA) with the genotype and the number of AC parents as factors and with age and sex as covariates was used to examine effects of the OXTR genotype, the number of AC parents, and their interaction on the TCI scores. When effects of interaction between the genotype and the number of AC parents on the TCI score were significant, post‐hoc one‐way ANCOVA was used for examinations of the effects of the number of AC parents on its score, separately in the A allele carriers and in the A allele noncarriers. The *χ* ^2^ test was used for comparison of the number of AC parents between the A allele carriers and the A allele noncarriers. *p‐*Values <.05 were inferred as significant.

## 3 RESULTS

Table 1 presents characteristics, TCI scores, OXTR genotype of the participants, and numbers of AC parents. The genotype distribution was in the Hardy–Weinberg equilibrium (*p* = .409).

**TABLE 1:** Characteristics, Temperament and Character Inventory (TCI) scores, oxytocin receptor (OXTR) genotype of the participants, and the number of affectionless control (AC) parents

Age, years	23.7 ± 3.1
Male/female, *n*	305/191
TCI, score	
Novelty seeking	20.8 ± 5.0
Harm avoidance	19.8 ± 6.0
Reward dependence	15.0 ± 3.7
Persistence	4.2 ± 1.8
Self‐directedness	27.9 ± 6.2
Cooperativeness	27.9 ± 5.5
Self‐transcendence	8.0 ± 4.2
OXTR rs53576, *n*	
G/G	60
G/A	237
A/A	199
Number of AC parents, *n*	
0	277
1	122
2	97

Table 2 presents the results of comparison of the number of AC parents between the A allele carriers and the A allele noncarriers. No significant difference was found in the number of AC parents between the two genotype groups (Table 2).

**TABLE 2:** Results of two‐way analysis of covariance for effects of the oxytocin receptor (OXTR) genotype and the number of affectionless control (AC) parents on the Temperament and Character Inventory (TCI) scores

	A allele noncarriers of the rs53576			A allele carriers of the rs53576			Main effect, *p*		
	No parent with AC	One parent with AC	Two parents with AC	No parent with AC	One parent with AC	Two parents with AC	OXTR genotype	No. of AC parents	Interaction, *p*
Novelty seeking	20.7 ± 5.0	21.5 ± 3.5	21.3 ± 5.2	20.8 ± 5.1	20.7 ± 5.1	20.9 ± 4.9	.660	.879	.865
Harm avoidance	20.8 ± 4.8	18.3 ± 6.6	20.0 ± 8.4	18.7 ± 5.9	20.8 ± 6.1	21.5 ± 5.7	.594	.440	.028
Reward dependence	15.6 ± 3.1	14.1 ± 3.6	14.3 ± 2.8	15.4 ± 3.8	14.6 ± 3.5	14.6 ± 3.7	.913	.239	.843
Persistence	4.3 ± 1.6	4.3 ± 2.0	4.9 ± 1.9	4.4 ± 1.8	4.0 ± 1.8	3.9 ± 1.8	.125	.689	.139
Self‐directedness	30.1 ± 5.9	26.3 ± 6.3	28.2 ± 5.4	28.9 ± 5.6	27.2 ± 6.6	25.3 ± 6.8	.295	.009	.327
Cooperativeness	28.7 ± 4.0	26.6 ± 4.6	26.3 ± 4.4	28.6 ± 5.8	27.2 ± 5.3	27.3 ± 5.1	.630	.156	.934
Self‐transcendence	8.0 ± 4.2	7.0 ± 3.2	10.0 ± 5.8	8.3 ± 4.4	7.4 ± 4.0	7.9 ± 3.7	.405	.104	.255

*Note*: Figures on the table except the main effect and interaction show mean scores ± SD. *p*‐Values were calculated using two‐way analysis of covariance with the genotype and the number of AC parents as factors and with age and sex as covariates.

**FIGURE 1:**
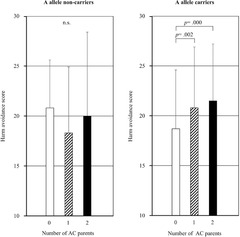
Effects of the number of affectionless control (AC) parents on scores of harm avoidance in the A allele noncarriers (left) and the A allele carriers (right). Differences in harm avoidance scores were tested by analysis of covariance followed by LSD tests

Abbreviations:

Table 3 presents the results of the two‐way ANCOVA for the effects of the OXTR genotype and the number of AC parents on the TCI scores. Two‐way ANCOVA showed significant interaction effects of the genotype and the number of AC parents on the scores of harm avoidance (*p *= .028), whereas significant main effects of the OXTR genotype were not found on any TCI scores (Table 3). Post‐hoc analysis showed that, in A allele carriers, the scores of harm avoidance were lower for participants with no parent with AC than for participants with one parent with AC (*p* = .002) and for participants with two parents with AC (*p* = .000) (Figure 1). No similar association was observed for A allele noncarriers (Figure 1).

**TABLE 3:** Number of affectionless control (AC) parents between the A allele carriers and the A allele noncarriers

	No parent with AC	One parent with AC	Two parents with AC
OXTR genotypes*			
A allele noncarriers, no. (%)	32 (53.3)	16 (26.7)	12 (20.0)
A allele carriers, no. (%)	245 (56.2)	106 (24.3)	85 (19.5)

Abbreviation: OXTR, oxytocin receptor.

* No significant difference in the number of AC parents between the two genotype groups.

(Yate's χ^2^ = 0.203, df = 2, *p *= .903).

## 4 DISCUSSION

For this study, significant interaction effects between the OXTR polymorphism and the number of AC parents on the scores of harm avoidance were found, with no main effect of genotype on any TCI dimension. Furthermore, scores of harm avoidance became higher in a stepwise manner with respect to the increase of the number of AC parents for A allele carriers, although no similar association was found for A allele noncarriers. It is suggested that high scores of harm avoidance are described as doubtful, pessimistic, shy, and fatigable, and that high harm avoidance is related to depression and anxiety disorders (Cloninger et al., 1994). The OXTR genotype of the participants was not associated with the number of AC parents, suggesting a lack of gene–environment correlation. Taken together, these results suggest that OXTR polymorphism does not affect the personality traits directly, but it might influence the characterization of harm avoidance by moderating susceptibility to AC parenting. These results are in line with those of earlier studies which suggest that the A allele is sensitive to negative parental environments such as maternal depression and low paternal care during childhood, which are related to increased susceptibility to depressive symptoms and behavioral problems (Choi et al., 2019; Kazantseva et al., 2020; Thompson et al., 2014). This report is the first of a study that specifically examines interaction effects between OXTR polymorphism and the environment on personality.

Three plausible mechanisms might be related to the present results. First, A allele carriers may have different levels of psychological resources or social support, which are related to harm avoidance. The results of earlier studies suggest that A allele carriers have lower psychological resources than A allele noncarriers (Saphire‐Bernstein et al., 2011). They do not benefit from social support leading to higher cortisol response to stress tests (Chen et al., 2011). Therefore, harm avoidance in A allele carriers might be increased in response to the number of parental AC because of less social support, whereas A allele noncarriers can buffer the effects of this dysfunctional parenting on harm avoidance using social support. Second, A allele carriers may be sensitive to negative environments which reduce hippocampal volume. Malhi et al. (2020) reported that the hippocampal volume becomes smaller in A allele carriers, but not in A allele noncarriers, from experiences of emotional trauma during childhood. Furthermore, Yamasue et al. (2008) described using magnetic resonance imaging that a hippocampus of small volume is related to high scores of harm avoidance. Therefore, the combination of A allele and AC parenting might promote reduction of hippocampal volume, leading to increased scores of harm avoidance. Third, changes in methylation levels of the OXTR gene induced by environments may be different between A allele carriers and noncarriers. Negative environments during childhood, for example, maltreatment (Fujisawa et al., 2019) and low maternal care (Unternaehrer et al., 2015), reportedly induce high methylation in several regions of the OXTR gene, which is linked to decreased OXTR expression (Kusui et al., 2001; Mamrut et al., 2013). Furthermore, the induction of the OXTR gene methylation is shown to be more apparent in A allele carriers than in A allele noncarriers (Reiner et al., 2015; Ziegler et al., 2015). Consequently, epigenetic mechanisms might be involved in the findings of the present study.

Several limitations exist for this study. First, the number of participants was not large. As a result, the number of A allele noncarriers was considerably small. Second, this study is a cross‐sectional study design, that is, the TCI and the PBI were assessed simultaneously, suggesting that the causative relation remains unclear. Third, the study participants were all Japanese. Therefore, these findings might be difficult to extend to other ethnic groups because of considerable ethnic diversity in allele frequencies of OXTR polymorphism (Kim et al., 2010).

## 5 CONCLUSION

The results of the present study suggest that the OXTR polymorphism influences the characterization of harm avoidance by moderating susceptibility to AC parenting.

## CONFLICT OF INTEREST

The authors declare no conflict of interest.

## AUTHOR CONTRIBUTIONS

Keisuke Noto designed the study, collected and analyzed the data, and wrote the first draft of the manuscript. Akihito Suzuk collected and analyzed the data and provided comments to the manuscript. Toshinori Shirata, Yoshihiko Matsumoto, Haruka Muraosa, Kaoru Goto, and Koichi Otani collected the data and provided comments to the manuscript. All authors read and approved of the final manuscript.

## FUNDING INFORMATION

Japan's Ministry of Education, Culture, Sports, Science and Technology.

### PEER REVIEW

The peer review history for this article is available at https://publons.com/publon/10.1002/brb3.2393.

## Data Availability

Datasets are available from the corresponding author on reasonable request.
